# Changes in the management of urinary tract infections in women: impact of the new recommendations on antibiotic prescribing behavior in France, between 2014 and 2019

**DOI:** 10.1186/s12913-021-06653-4

**Published:** 2021-06-28

**Authors:** Arthur Piraux, Sébastien Faure, Kurt G. Naber, Jakhongir F. Alidjanov, Aline Ramond-Roquin

**Affiliations:** 1 Univ Angers, Inserm, CNRS, MINT, SFR ICAT, 16 boulevard Daviers, F-49000 Angers, France; 2 Department of Urology, Technical University of Munich, Munich, Germany; 3 Department of Urology, Pediatric Urology and Andrology, Justus-Liebig University of Giessen, Giessen, Germany; 4 Faculté de Santé, Département de Médecine Générale, Univ Angers, F-49000 Angers, France; 5 Univ Angers, Univ Rennes, EHESP1, Inserm, IRSET-ESTER, SFR ICAT, F-49000 Angers, France

**Keywords:** Urinary tract infection, Antimicrobial resistance, Antibiotic consumption, Guideline adherence, Prescriber-pharmacist collaboration, Educational intervention, Antibiotic resistance

## Abstract

**Background:**

Urinary tract infections (UTIs) are among the most common infections that require antibiotic therapy. In December 2015, new guidelines for UTI management were published in France with the aim of reducing antibiotic misuse and the risk of antimicrobial resistance.

**Objectives:**

To analyze changes in antibiotic prescribing behavior for acute uncomplicated UTI in women in France from 2014 to 2019.

**Methods:**

Retrospective study using data extracted from the medico-administrative database ‘OpenMedic’ that is linked to the French National Health Data System and collects data on the reimbursement of prescribed drugs. The analyses focused on the number of boxes of antibiotics delivered by community pharmacies, the molecule class, and the prescriber’s specialty.

**Results:**

Overall, antibiotic dispensing by community pharmacies increased by 2% between 2014 and 2019, but with differences in function of the antibiotic class. The use of antibiotics recommended as first-line and second-line treatment increased (+ 41% for fosfomycin and + 7430% for pivmecillinam). Conversely, the dispensing of lomefloxacin and norfloxacin decreased by 80%, and that of ciprofloxacin by 26%. Some antibiotics were mostly prescribed by general practitioners (lomefloxacin, pivmecillinam) and others by secondary care physicians (ofloxacin). Dispensing increased for antibiotics prescribed by secondary care physicians (+ 13% between 2014 and 2019) and decreased for antibiotics prescribed by GPs (− 2% for the same period).

**Conclusion:**

These data suggest that the new recommendations are followed, as indicated by the increased prescription of fosfomycin and pivmecillinam and decreased prescription of fluoroquinolones. However, the efficient transmission and implementation of new recommendations by practitioners requires time, means and dedicated tools.

**Supplementary Information:**

The online version contains supplementary material available at 10.1186/s12913-021-06653-4.

## Background

Urinary tract infections (UTIs) are among the most common bacterial infections, particularly in women [1–3]. Yet, UTI incidence is difficult to estimate because this is not a reportable disease in many countries and in primary care settings the diagnosis may not be always confirmed by urine testing. Previous studies found that about 10% of > 18-year-old women presented UTI symptoms in the 12 months preceding the survey, and that approximately 50% of women had at least one episode of cystitis during their life [1, 4]. In France, 4 to 6 million people have UTI each year. It is one of the main reasons for consulting a general practitioner (GP) [3, 5, 6].

UTIs, particularly acute uncomplicated cystitis, are usually treated with short-course antibiotic regimens. However, bacterial resistance to some antibiotics currently used for managing community-acquired UTIs has been detected in primary care settings and hospitals worldwide [7–9]. Antimicrobial resistance (AMR) is considered one of the most important threats to modern medicine by the World Health Organization (WHO) [8, 10, 11]. According to a recent WHO report, bacterial resistance has been found in almost 40% of human infections in some developed countries [12]. In the case of UTIs, antibiotic resistance is associated with delayed symptom resolution, and might cause pyelonephritis [13, 14]. Therefore, to limit AMR increase, recommendations on UTI management are regularly updated by the health authorities and scientific societies [7, 15, 16]. For instance, the current guidelines indicate that in women with uncomplicated UTI, fluoroquinolones (e.g. lomefloxacin and norfloxacin) should only be prescribed as last-line treatment [15, 17]. In France, the *Société de pathologie infectieuse de langue française* (French infectious disease society) and the *Haute autorité de la santé* (French national health authority) updated their guidelines on UTI management in December 2015 and now recommend a single dose of fosfomycin for acute uncomplicated cystitis (Table 1). Moreover, to limit the use of lomefloxacin and norfloxacin, the French authorities decided to stop their reimbursement from June 2019. Additionally, several public health campaigns have been deployed to make people aware of AMR risk and to explain the correct use of antibiotics [10, 20–22].

**Table 1:** French guidelines for the treatment of acute uncomplicated cystitis

	Before December 2015 [18]	From December 2015 [7, 19]
**First line**	Fosfomycin as a single dose	Fosfomycin as a single dose
**Second line**	Nitrofurantoin for 5 days Fluoroquinolone^a^ as single dose (*ciprofloxacin, ofloxacin*) Fluoroquinolone^a^ for 3 days (*ciprofloxacin, lomefloxacin, norfloxacin, ofloxacin*)	Pivmecillinam for 5 days
**Third line**		Nitrofurantoin for 5 days Fluoroquinolone^a^ as single dose (*ciprofloxacin, ofloxacin*)

^a^ The use of fluoroquinolone is not indicated if another quinolone has been taken in the previous six months, regardless of the indication

The objective of this study was to analyze how antibiotic prescriptions for the management of acute uncomplicated UTI in women changed in France between 2014 and 2019 to determine whether and to what extent these new guidelines have been implemented. Indeed, to reduce AMR, it is important to study the impact of health policies, recommendations, and awareness campaigns on the physicians’ prescribing behaviors.

## Method

### Study design

Retrospective study based on data extracted from a medico-administrative database linked to the *Système National Interrégimes de l’Assurance Maladie* (French National Health Insurance System).

### Choice of database

The ‘OpenMedic’ database collects data from the *Système National des données de santé* (National Health Data System) and was selected because it provides exhaustive information on drugs (Anatomical Therapeutic Chemical, ATC, classification) dispensed by community pharmacies to the entire French population since 2014 [23]. This database, available online and with an open license, allows determining the origin of drug prescriptions presented to community pharmacies and investigating drug utilization in France (reimbursed and non-reimbursed drugs). These data are collected by the *Caisse Nationale de l’Assurance Maladie* (French National Health Insurance System), in charge of medication reimbursement in France.

### Choice of antibiotics

Only data on antibiotics included in the 2015 guidelines on UTI management were selected from the ‘OpenMedic’ database (Table 1) [3, 7, 19, 24]. Some of these antibiotics are only used for treating acute cystitis (fosfomycin, nitrofurantoin, and lomefloxacin), while others are prescribed for UTI management in general (pivmecillinam and norfloxacin) and also for other infection types (ciprofloxacin, ofloxacin and norfloxacin). To limit the lack of information on the indication in the database, only oral antibiotics were selected (ear and eye drops, and injectable forms were removed). All antibiotics dispensed by French pharmacies are only available as prescription drugs and not as over-the-counter drugs.

### Inclusion criteria and study variables

As the OpenMedic database does not give the reason (i.e. diagnosis) that led to the antibiotic prescription, some of the variables available in this database (Additional file 1: Table 1) were selected as inclusion criteria to obtain a patient typology as close as possible to that of a woman with uncomplicated UTI:

- Female sex (exclusion of men and unknown sex);
- Age between 20 and 59 years (age > 65 years may be a risk factor for UTI complications, according to the French recommendations [19]);
- Living in France;
- The selected antibiotics were identified using the fifth level (i.e. chemical substances) of the ATC classification and their presentation ID code (to select only oral antibiotics).

Among the 25 different types of prescribers available in this database (Additional file 1: Table 1), two categories were selected: general practitioners (GPs) and secondary care physicians (including emergency services and physicians/GPs working in private clinics and residential care homes). These physicians are the most accessible and appropriate for the management of uncomplicated UTIs; these two categories alone account for more than 90% of all prescriptions.

### Statistical analysis

The French healthcare system and particularly the modalities for prescribing and dispensing drugs did not allow us to use the daily defined dose to describe antibiotic consumption. Therefore, we used the number of boxes of a specific antibiotic dispensed by community pharmacies per year. Indeed, the database counts the deliveries per patient over a year in a binary way (no = 0/yes ≥1), regardless of the number of boxes dispensed to that patient during that year (1 episode = 1 delivery / 5 episodes = 1 delivery). Moreover, the delivery of two boxes of the same antibiotic type does not imply that a patient took all the tablets contained in the two boxes (e.g. pivmecillinam). Similarly, the delivery of four boxes of the same molecule does not imply that the four boxes were used to treat a single episode (e.g. fosfomycin).

The various analyses focused on the number of boxes of the main antibiotics indicated for UTI delivered by community pharmacies for each year and the prescriber type.

## Results

### Changes in antibiotic delivery from 2014 to 2019

**Fig. 1:**
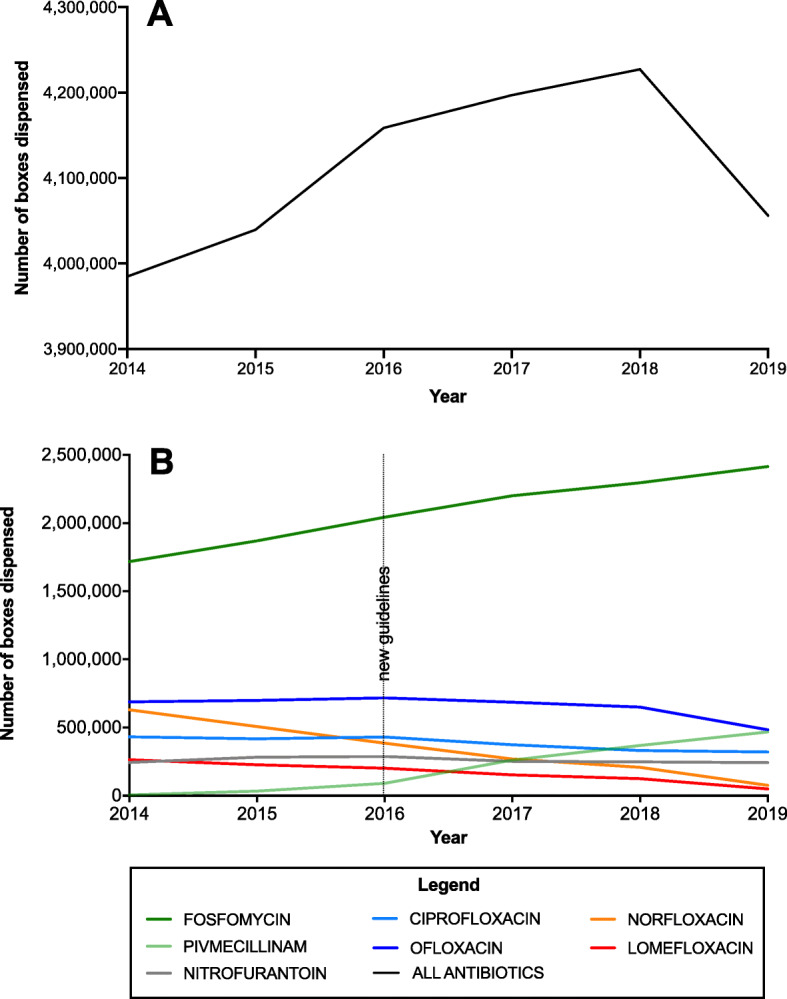
Changes in antibiotic consumption, in France, between 2014 and 2019. **a** Overall consumption (assessed as number of boxes dispensed by community pharmacists per year) of the main antibiotic classes used for UTI management (fosfomycin, pivmecillinam, nitrofurantoin, ciprofloxacin, ofloxacin, norfloxacin, and lomefloxacin); **b** Temporal changes in the delivery by community pharmacists of the indicated antibiotics used for acute uncomplicated UTI

Overall, the number of antibiotic boxes commonly used for UTI treatment and dispensed by community pharmacies (Fig. 1A) increased by 6% from 2014 to 2018 (3,984,834 boxes prescribed in 2014 and 4,227,236 in 2018), and then slightly decreased from 2018 to 2019 (4,056,035 prescribed boxes in 2019), resulting in a 2% increase during the study period. However, this relative stable trend hid differences among antibiotic classes (Fig. 1B). Specifically, the prescription of pivmecillinam, an antibiotic that is now recommended as second-line treatment, progressively and rapidly increased (+ 434% in 2015, + 4107% in 2017, + 7430% in 2019; 6000 boxes were delivered in 2014 and more than 467,000 in 2019). The delivery of fosfomycin (the first-line drug for uncomplicated UTI) also progressively increased, but more slowly (+ 9% in 2015, + 28% in 2017, and + 41% in 2019). On the other hand, the delivery of lomefloxacin and norfloxacin strongly decreased, particularly in 2019 (− 82% and − 88%, respectively, compared with 2014). Ciprofloxacin and ofloxacin consumption showed a smaller decrease (− 26% and − 30%, respectively, in 2019 compared with 2014), while nitrofurantoin delivery remained stable (− 0.6%) during the study period.

GPs were the most common prescribers of antibiotics for UTI management (76% of all antibiotic prescriptions for UTI; approximately 4.2 million antibiotic boxes per year counted by the French National Health Insurance system), followed by secondary care physicians (16% of all antibiotic prescriptions). These two categories alone were responsible for more than 92% of all prescriptions.

### Changes in antibiotics delivered depending on the Prescriber’s type

**Fig. 2:**
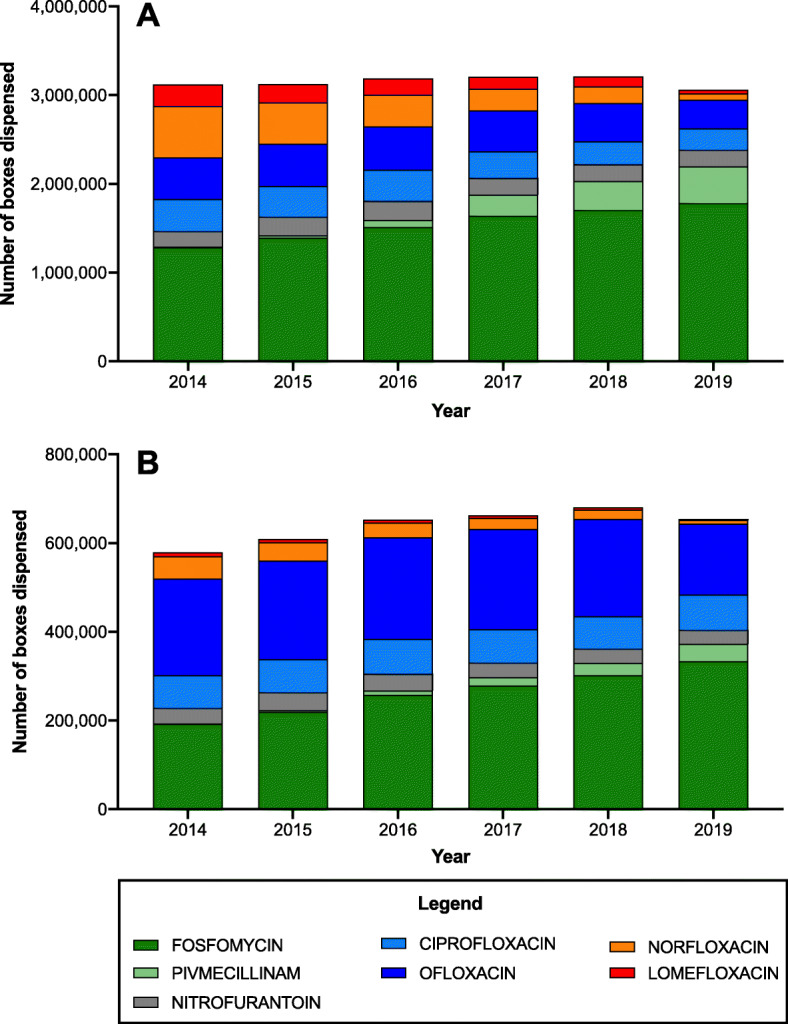
Changes in antibiotics delivered by community pharmacies in France, between 2014 and 2019, according to the prescriber type: (**a**) General practitioners; (**b**) Secondary care physicians

To evaluate the implementation of the 2015 UTI recommendations, the prescribing patterns of GPs and secondary care physicians were investigated (Fig. 2). During the study period, their prescribing behavior changed in line with the national recommendations, but some differences could be observed between GPs (Fig. 2A) and secondary care physicians (Fig. 2B).

Fosfomycin remained the most prescribed molecule, and its delivery by community pharmacies increased over time (+ 39% for GPs and + 74% for secondary care physicians between 2014 and 2019). Conversely, the prescription of pivmecillinam, which is now recommended as a second-line treatment, increased more among GPs than secondary care physicians (respectively + 8207% and + 3913%). However, nitrofurantoin prescription frequency did not change in both groups. Ciprofloxacin and ofloxacin were preferentially prescribed by secondary care physicians than by GPs. In 2019, these two molecules appeared in 37% of secondary care physicians’ prescriptions and in 18% of GPs’ prescriptions. Conversely, lomefloxacin and norfloxacin, which are no longer reimbursed since June 2019, were more frequently prescribed by GPs than secondary care physicians. In 2014, they represented 27 and 9% of all molecules prescribed by GPs and secondary care physicians, respectively. In 2019, they represented only 4 and 2% of all prescriptions by GPs and secondary care physicians, respectively.

Overall, the main trends observed in Fig. 1 (increase in 2018 before a decrease in 2019) were also detected for the antibiotic prescribing behaviors of GPs (+ 3% between 2014 and 2019), but not of secondary care physicians (+ 18% for the same period).

## Discussion

### Main results

The OpenMedic data, which reflect the antibiotic delivery by community pharmacies, showed variations according to the molecule class and the prescriber considered. We observed the emergence of pivmecillinam, an increase of fosfomycin, and a large decrease of norfloxacin and lomefloxacin prescriptions. This very large decrease, more than 80%, for lomefloxacin and norfloxacin is an accomplishment on its own. Although the non-reimbursement of these molecules was implemented only at the end of the study period (in 2019), their prescription progressively decreased since the beginning of the study (in 2014). As the French national health authority opinion to end their reimbursement was issued in February 2017, we can hypothesize that these two years allowed prescribers to be informed about the decision, and to get acquainted with the new recommendations [25, 26]. In addition, in France, most patients do not need to pay for their medications at the community pharmacy because the Health Insurance System pays directly the pharmacists. Therefore, they may not understand and accept to pay for an antibiotic prescribed by a physician for UTI. As a consequence, they may refuse to buy the antibiotic, they may go to another physician, or they may ask the pharmacist to contact the prescriber in order to change the prescribed antibiotic molecule [27].

Some molecules were more specific to a specific prescriber. For example, pivmecillinam was prescribed mainly by GPs, and ofloxacin by secondary care physicians. In 2019, about 16% of fosfomycin prescriptions were by secondary care physicians who are working in centers in which the resources and technical facilities are not designed to manage acute uncomplicated UTI. This could mirror the increase in the use of the emergency department even for minor health problems, particularly during weekends and weeknights (i.e. when GPs are often not on duty in France) [5, 28, 29]. Indeed, such prescriptions were mainly issued in a hospital, but the antibiotic was dispensed by a community pharmacy.

The finding about the high percentage of fluoroquinolone (ciprofloxacin, ofloxacin) prescriptions by secondary care physicians should also be considered with caution because they are prescribed for many infections that are often more serious than UTI. Moreover, the recommendations for other common infectious diseases were not changed during the study period. For instance, the latest recommendations for adult lower respiratory tract infections were published in 2010 [30] and for upper respiratory tract infections in 2011 [31]. Conversely, the proportion of pivmecillinam prescriptions increased in both groups but much more in the GP group. Overall, these differences can be explained by the fact that uncomplicated UTIs are, in theory, managed by GPs, while complicated infections are treated in hospital and do not have the same recommendations and treatments [7, 15].

### Impact of expert recommendations and health policies

The study by Grol et al. focused specifically on the most effective ways to implement new scientific evidence in the daily medical practice [32]. Many tools are available for policymakers and practitioners, but no method seems to perform better than the others. Therefore, the choice of tool should be based on the specific environment and the health professionals targeted by the new recommendations/changes. Moreover, such tool should also allow regularly assessing the impact of the new measures.

The year 2016, when the new recommendations were implemented, was characterized by the strong increase in the delivery of pivmecillinam, which officially entered the therapeutic arsenal, and the significant decrease of ciprofloxacin. However, as the physicians’ adherence to recommendations is poor, policymakers should combine the publication of new guidelines with interventions to convince prescribers [24, 33–37]. For example, in England, to reduce inappropriate UTI management, and to limit the risk of trimethoprim resistance, the National Health Service introduced the ‘Quality Premium’ program in 2016 with the aim of reducing trimethoprim prescriptions through financial incentives [38]. Similarly, the French health authorities concluded that lomefloxacin and norfloxacin have insufficient medical value and decided to stop their reimbursement at the beginning of 2019 to limit their use. This measure seems to be useful, as indicated by the very strong decrease in their delivery in 2019 and was supported by a broad-based communication (scientific congresses, websites and newsletters) to promote the new recommendations. For example, some very synthetic ‘memos’, including the key messages of the new recommendations were proposed [19].

The Infectious Diseases Society of America introduced new guidelines for UTI treatment in 2011 [39]. To measure compliance with these new recommendations, a retrospective analysis was carried from 2009 to 2013. The authors observed a positive change in the choice of antibiotic class. Conversely, the new guidelines did not seem to improve the inappropriate duration of antibiotic therapy. According to the authors, antimicrobial stewardship (AMS) interventions are needed to improve antibiotic prescribing for uncomplicated UTIs. AMS initiatives are regularly deployed in hospitals to prevent AMR. An AMS strategy implemented in emergency departments in Ohio, United States of America, was based on a two-step intervention [40]: i) an electronic order set based on the most recent guidelines for UTI treatment, with a financial incentive for its use, followed by ii) an audit and feedback. The first step of this AMS intervention led to a significant increase in the adherence to guidelines (from 44 to 68%) that continued to increase to 82% after the second step.

### How to improve the implementation of recommendations

Besides the application of recommendations in terms of drug choice, it is important also to use the most adapted care pathway. Indeed, acute cystitis should be managed only by GPs and community pharmacists. However, uncomplicated UTIs are still a frequent reason to visit emergency departments [28, 41].

Moreover, all physicians, and particularly GPs should be aware of the need to comply with the latest recommendations to reduce AMR [42]. One of the keys to the successful implementation of new recommendations is knowledge transfer, and many solutions are already available [43]. For example, a computerized decision support platform can be a tool to translate complex healthcare knowledge into everyday practice [44]. A study evaluated the impact of a mobile phone application to increase guideline adherence by prescribers caring for inpatients with community-acquired pneumonia or urinary tract infections [45]. The authors observed an increase in the adherence to the antibiotic guidelines, but only for pneumonia management. Nonetheless, this type of tool seems to work for prescribers. Indeed, 145 health workers downloaded the application during the study period, and more than 3000 downloads were recorded several months later. In France, several quick and simple computer tools (e.g. Antibioclic© and Vidal Recos® [46, 47]) are already available to find the recommended care pathway and most suitable treatment.

Some barriers need to be overcome. Communication, especially between GPs and institutions, is an important point [48]. Scientific societies and institutions have to work with physicians to make these recommendations more “user-friendly”. Physicians could be included in the groups involved in guideline drafting to make them accessible to all and as close as possible to the real practice conditions. All the main concerned parties (clinicians, researchers, knowledge users, and institutions) should be brought together to identify common challenges and success factors for the implementation of a new program [49].

More than insufficient knowledge, lack of agreement with the recommendations and lack of applicability seem to be the main barriers to guideline adherence [50]. To overcome these barriers, education sessions could be proposed where small groups of GPs (or other healthcare professionals) can analyze their current practices and find ways to include the new recommendations [37, 40]. This approach is useful, but possibly not in the long term [51].

Several studies have assessed the impact of AMS in hospital settings, but very few in communities [52–55]. A recent article examined specifically AMS interventions in the community [56], and again, found that education-focused interventions seem to be efficient to limit AMR. They also highlighted the lack of research on this topic in communities.

A systematic review in English primary care tried to identify ways to optimize AMS interventions [57]. The authors identified 41 types of influences on antibiotic prescribing that were categorized in six theoretical domains frameworks. To improve guideline implementation, they suggested, for example, electronic decision support tools, workshops on antibiotic prescribing, and implementing evidence-based practice protocols. They stressed that these types of AMS interventions should be implemented also in other primary care settings, such as community pharmacies.

Moreover, the collaboration between physicians and community pharmacists could be strengthened, particularly when they work in the same community. The pharmacist could contribute to optimize the prescription of antibiotics for UTIs and help to limit unnecessary antibiotic exposure [58–60]. Different quality indicators (e.g. dosage, duration, antibiotic/antibiogram suitability) were improved after a pharmacist’s intervention (> 96% of conformity for treatment duration and 98% for posology) [58].

### Limitations

The lack of knowledge on the diagnosis is one of the biggest limits of this study. Despite the inclusion criteria based on sex and age, we cannot be sure that the delivered antibiotics were prescribed for UTI, although some of them (e.g. fosfomycin) should be used only for acute uncomplicated UTI in women. Moreover, the lack of information on the diagnosis did not allow checking the relevance of the antibiotic prescriptions. Therefore, antibiotics that can be used also for other indications were restricted to oral forms only, by eliminating all deliveries of ocular, auricular and injectable formulations.

The analysis of the number of boxes dispensed did not allow knowing the number of UTI episodes per year. Some treatments, for an episode, requires two boxes of antibiotics (pivmecillinam) and in other cases, the antibiotic can be used continuously for the prevention of recurrent cystitis (fosfomycin). Therefore, this analysis was based on an exhaustive result. The number of consumers, also available in this database, induced other biases (if a woman had three UTI episodes in the same year and was treated with the same antibiotic, she would have been counted only as one consumer). Data on the daily defined doses instead of the number of boxes would improve accuracy, and also allow comparing the French data with those of other countries.

Only the antibiotics recommended for UTI were selected, but others are prescribed to treat UTI (e.g. trimethoprim-sulfamethoxazole). Moreover, a drug dispensed in a pharmacy may be not taken by the patient for different reasons, including negative results of the urine culture. Finally, the non-reimbursement of lomefloxacin and norfloxacin from 2019 may have led to underreporting of their dispensing (if pharmacists do not submit the invoice to Health Insurance).

Another limitation concerns the database. As variables are limited, particularly the age groups (0–20 years, 20–60 years, more than 60 years), we could not assess differences in antibiotic prescriptions to women of different ages. It would be relevant to have narrower age classes for future analyses.

### Future research

It should be important to code each medical procedure to allow a more detailed and objective analysis of antibiotic prescription practices and adherence to recommendations [14, 38, 61]. The knowledge of the diagnosis should also be useful for community pharmacists to check the antibiotic prescription appropriateness.

Besides, it would be interesting to follow a cohort of patients to analyze their care pathway (category of practitioners, time required to receive the diagnosis and treatment, etc.) to identify and propose strategies to improve adherence to healthcare guidelines.

## Conclusion

To limit AMR, the French authorities reviewed their recommendations for UTI management in 2015 and fluoroquinolones are now only recommended as a last resort for uncomplicated infection. The new recommendations seem to be increasingly followed, on the basis of the changes in the delivery of antibiotics recommended for UTI treatment: an increase of fosfomycin and pivmecillinam, and a decrease of fluoroquinolones.

However, there is still place for improvement. Health policymakers must encourage and promote adherence to such recommendations. Financial motivations, audits and feedback, educational interventions, implication of primary healthcare professionals, prescriber-pharmacist collaborations are among the many resources available to help physicians. These approaches are particularly useful in primary care, where most patients go for UTI and where the potential is high to significantly limit AMR.

## Supplementary Information

**Additional file 1: Table 1**: Variables available in the ‘OpenMedic’ database*.

## Data Availability

The datasets analyzed in the present study are available in the ‘OpenMedic’ database that collects data from the *Système National des données de santé* (National Health Data System), https://www.data.gouv.fr/fr/datasets/open-medic-base-complete-sur-les-depenses-de-medicaments-interregimes/

## Abbreviations

AMR: Antimicrobial resistance
AMS: Antimicrobial stewardship
ATC: Anatomical Therapeutic Chemical
GP: General practitioner
UTI: Urinary tract infections
WHO: World Health Organization
