# Supplementary material for: Changes in the management of urinary tract infections in women: impact of the new recommendations on antibiotic prescribing behavior in France, between 2014 and 2019
Source: BMC Health Serv Res. 2021 Jun 28;21:612. doi: 10.1186/s12913-021-06653-4 (PMC8240268; doi:10.1186/s12913-021-06653-4)
Supplement: Supplementary file 1 — Additional file 1: Table 1: Variables available in the ‘OpenMedic’ database*. [file 12913_2021_6653_MOESM1_ESM.docx]

Changes in the management of urinary tract infections in women: impact of the new recommendations on antibiotic prescribing behavior in France, between 2014 and 2019

Arthur PIRAUX, PharmD-PhD student^1^; Sébastien FAURE PharmD-PhD^1^; Kurt G. NABER^2^; Jakhongir F. ALIDJANOV^3^; Aline RAMOND-ROQUIN Md-PhD^4,5^

1. Univ Angers, Inserm, CNRS, MINT, SFR ICAT, F-49000 Angers, France

2. Department of Urology, Technical University of Munich, Munich, Germany

3. Department of Urology, Pediatric Urology and Andrology, Justus-Liebig University of Giessen. Giessen, Germany

4. Univ Angers, Univ Rennes, EHESP1, Inserm, IRSET-ESTER, SFR ICAT, F-49000 Angers, France

5. Département de médecine de famille et de médecine d'urgence, University of Sherbrooke, Quebec, Canada

**Appendix Table 1: Variables available in the ‘OpenMedic’ database***

| **Variable** | **Category** | **Meaning** |
| --- | --- | --- |
| **Age** | 0 | 0-19 YEARS |
|  | 20 | 20 - 59 YEARS |
|  | 60 | 60 YEARS AND MORE |
|  | 99 | AGE UNKNOWN |
| **Sex** | 1 | MAN |
|  | 2 | WOMAN |
|  | 9 | UNKNOWN VALUE |
| **Prescriber** | 1 | GENERAL PRACTITIONER |
|  | 2 | ANESTHESIOLOGIST/INTENSIVE CARE |
|  | 3 | CARDIOVASCULAR DISEASE SPECIALIST |
|  | 4 | SURGEON |
|  | 5 | DERMATOLOGIST |
|  | 6 | RADIOLOGIST |
|  | 7 | GYNECOLOGIST/OBSTETRICIAN |
|  | 8 | GASTROENTEROLOGIST AND HEPATOLOGIST |
|  | 9 | INTERNAL MEDICINE DOCTOR |
|  | 11 | OTORHINOLARYNGOLOGIST |
|  | 12 | PEDIATRICIAN |
|  | 13 | PULMONOLOGIST |
|  | 14 | RHEUMATOLOGIST |
|  | 15 | OPHTHALMOLOGIST |
|  | 17 | PSYCHIATRIST |
|  | 18 | ORAL MEDICINE SPECIALIST |
|  | 31 | PHYSIATRIST |
|  | 32 | NEUROLOGIST |
|  | 35 | NEPHROLOGIST |
|  | 37 | PATHOLOGIST |
|  | 38 | CLINICAL LABORATORY DIRECTOR |
|  | 42 | ENDOCRINOLOGIST |
|  | 90 | SECONDARY CARE PHYSICIANS |
|  | 98 | OTHER COMMUNITY PRESCRIBERS (Dentists, Paramedics, Laboratories, Midwives…) |
|  | 99 | UNKNOWN VALUE |

*Variables selected for this study are highlighted.
